# 

**Published:** 1876-09

**Authors:** 


### Contents of September Number.

### ORIGINAL.

ART. I.—The Treatment of the Pedicle in Ovariotomy. By F. W. Strange, M. R. C. S., Eng.... .................................. 41
ART. II.—Malignant Disease of the Tonsil. Removal by Galvano-Cautery. By E. N. Brush, M. D.................................. 46

### PROCEEDINGS OF MEDICAL SOCIETIES.

The International Medical Congress........................................ 48

### EDITORIAL.

The International Medical Congress. ................................ ........ 81

